# Collagen-Based 3D Scaffolds from Sea Urchin Food Waste for Skeletal Muscle Tissue Engineering

**DOI:** 10.3390/ani16030512

**Published:** 2026-02-05

**Authors:** Eylem Emek Akyürek, Luca Melotti, Martina Erba, Anna Carolo, Giordana Martinelli, Margherita Roncoroni, Stefania Marzorati, Marco Patruno, Michela Sugni, Roberta Sacchetto

**Affiliations:** 1 Department of Comparative Biomedicine and Food Science, University of Padova, Viale dell’Università 16, Legnaro, 35020 Padova, Italy; eylememek.akyurek@unipd.it (E.E.A.); luca.melotti@unipd.it (L.M.); martina.erba@phd.unipd.it (M.E.); anna.carolo@unipd.it (A.C.); marco.pat@unipd.it (M.P.); 2 Department of Environmental Science and Policy, University of Milan, Via Celoria 26, 20133 Milano, Italy; giordana.martinelli@unimi.it (G.M.); margherita.roncoroni@unimi.it (M.R.); stefania.marzorati@unimi.it (S.M.); michela.sugni@unimi.it (M.S.)

**Keywords:** marine collagen-based scaffold, 3D muscle construct, skeletal muscle tissue models

## Abstract

Preclinical investigations involving in vivo animal studies, are considered a crucial and mandatory step in pharmacological industry regulations. In recent years, as the ethical concerns associated with animal experimentation are becoming more relevant, substantial research efforts have been directed towards the development of novel in vitro methodological approaches aimed at minimizing the utilization of animals. Among those, three-dimensional (3D) cell culture systems have become one of the most promising substitutes for animal models. Skeletal muscle can be affected by genetic disorders and injuries. In this study, two types of 3D scaffolds (Coll and CollMA), prepared starting from a fibrillar collagen suspension extracted from sea urchin food waste, were used as platforms for the generation of 3D skeletal muscle tissue models. The scaffolds were cellularized with C2C12 myoblasts. As the culture period progressed, C2C12 cells gradually infiltrated and were viable throughout both scaffold types, establishing a multilayered cellular population. In the CollMA scaffold, the reciprocal Pax7/MyoD expression pattern suggests a progression of myoblasts towards myogenic commitment. Taken together, our results, although preliminary, suggest that sea urchin–derived collagen matrix represents a promising scaffold for skeletal muscle tissue engineering.

## Simple Summary

Skeletal muscle comprises around 40% of total body mass in mammals. It consists of bundles of muscle fibers surrounded by an extracellular matrix providing structural support, with collagen as the principal component. Several disorders can affect skeletal muscle, many being rare genetic diseases currently lacking specific therapies. Animal models are widely used in biomedical research and the pharmaceutical industry to study disease mechanisms and develop new treatments. However, there is a growing need for alternative approaches that reduce animal use while maintaining physiological relevance. In this context, the pharmaceutical sector is increasingly adopting the One Health framework, which recognizes the interconnection between human health, animal health, and environmental sustainability. This study proposes two 3D skeletal muscle tissue models. These collagen-based models mimic the muscle extracellular matrix using sea urchin collagen, supporting muscle cell growth, infiltration, and differentiation. In the future, such tissue models may enable patient-derived muscle constructs for evaluating precision and personalized therapies, reducing animal testing. Additionally, utilizing collagen from food industry waste aligns with circular economy principles.

## 1. Introduction

Drug development is a long and costly process in both human and veterinary medicine, as new pharmacologically active compounds require several years to progress from the bench to the bedside [1].

Following the identification of promising compounds, novel therapeutic molecules are entering clinical trials involving patients. Nevertheless, the first stage in evaluating the efficacy and biological effects of a drug candidate is provided by preclinical testing. These investigations encompass experimental protocols comprising both in vitro and in vivo studies.

In vitro studies, which mainly utilize monolayer cell cultures in plastic dishes, are fundamental for confirming biological mechanisms, assessing experimental settings, and testing therapeutic hypotheses. However, these techniques represent only the first step toward a cure.

In fact, preclinical investigations involving in vivo animal studies are considered a crucial and mandatory step in pharmacological industry regulations [2]. Moreover, they are deemed indispensable prior to initiating clinical trials on patients. In vivo studies are typically conducted on rodents (mainly mice) [3], which are the most used animal models for studying human diseases, in drug development research, and for preclinical evaluation of new therapeutic molecules. The mouse and human genomes exhibit a high degree of conservation, as 99% of mouse genes overlap with those of humans [4]; consequently, the mouse is widely regarded as the gold-standard animal model for human drug testing, despite several non-negligible interspecies differences.

However, the use of animals involves biological, technical constraints, and above all, ethical considerations. Furthermore, in recent decades, significant ethical concerns have been raised regarding the use of murine and other animal models in biomedical research. In recent years, significant efforts have been made by legislators from several countries to render enforceable and applicable the principles of the 3Rs (reducing, refining, and replacing), defined by Russell and Burch in 1959 [5].

While reduction (minimizing the number of animals utilized in experimental research) and refining (implementing techniques aimed at mitigating animal distress) are principles partially already adopted by a considerable number of nations worldwide, replacing remains the most ambitious objective to be accomplished. In recent years, however, as the ethical considerations associated with animal experimentation are becoming relevant, substantial research efforts have been directed towards the development of novel in vitro methodological approaches aimed at minimizing the utilization of animals.

Among those in vitro approaches, three-dimensional (3D) cell culture systems have become one of the most promising substitutes for animal models in the drug discovery pipeline and in the drug development process [6,7]. Particularly, over the past two decades, extensive investigations have been undertaken to engineer functional 3D matrices, denominated as scaffolds, in which to establish a cell culture system [8]. Due to physical and chemical interaction with cells, the scaffold matrix is characterized by the ability to recapitulate the organization and microarchitecture of living tissues and organs.

The World Health Organization estimates that approximately 1.7 billion individuals globally are affected by skeletal muscle conditions, most of them without a cure, characterized by progressive reduction in muscle functions, limiting daily living. For this reason, the development of biologically relevant 3D muscle models in vitro could be a valuable asset. Using a 3D construct that could resemble the native muscle tissue architecture and cell–cell interaction might serve as an optimal preclinical model for investigation, reducing the use of animals while acting as a useful tool in the tissue engineering and regenerative medicine field.

In the present study, using tissue engineering techniques, we propose two types of innovative 3D scaffolds composed of collagen derived from sea urchins [9,10,11]. The peculiarity of this collagen is that it is obtained and used in its fully native fibrillar form (i.e., preserving the supramolecular organization of collagen), avoiding any enzymatic or hydrolytic treatment, and allowing the maintenance of the natural glycosaminoglycan surface decoration of the fibrils [12]. This feature allows the production of mechanically resistant and more biomimetic scaffolds [9] which reproduce the natural extracellular environment with more fidelity, likely including bioactive and mechanical signals [13].

To mimic skeletal muscle architecture, these natural scaffolds have been subsequently re-cellularized with cells of muscle origin to generate tissue models with very high proximity to muscle tissue physiology.

## 2. Materials and Methods

### 2.1. Sea Urchin Waste Recovery

Sea urchin waste from the species Paracentrotus lividus of the Adriatic Sea was provided by local restaurants near the University of Milan. It was promptly frozen and stored at −20 °C until required. For collagen extraction, the waste was thawed, and the peristomial membranes were separated from the test.

### 2.2. Sea Urchin Collagen Extraction

Collagen was extracted using a protocol published by [12,14]. Briefly, the membranes were rinsed in artificial seawater and left overnight at RT in hypotonic solution (10 mM Tris, 0.1% EDTA, pH 8; Sigma-Aldrich, Merck KGaA, Darmstadt, Germany). Following several washes in phosphate-buffered saline (PBS; Sigma-Aldrich, Merck KGaA, Darmstadt, Germany), samples were left overnight at RT in decellularizing solution (10 mM Tris, 0.1% Sodium Dodecyl Sulphate, pH 8; Sigma-Aldrich, Merck KGaA, Darmstadt, Germany). Numerous washes in PBS were performed, and the disaggregating solution (0.5 M NaCl, 0.1 M Tris-HCl, pH 8.0, 0.1 M β-mercaptoethanol, 0.05 M EDTA; Sigma-Aldrich, Merck KGaA, Darmstadt, Germany) was added and left at RT for 5 days. All these steps were performed in stirring conditions. The resulting collagen suspension was filtered through a stainless-steel mesh filter and dialyzed first against 0.5 M EDTA for four hours and then against distilled water overnight to eliminate any residual β-mercaptoethanol. Collagen concentration (mg/mL) was measured by drying and weighing a known aliquot of the obtained collagen suspension. The final aqueous collagen suspension was stored at −80 °C until further use.

### 2.3. Production and Ultrastructure Characterization of Methacrylated Collagen Hydrogel (CollMA) and Standard Collagen (Coll) Scaffolds

A defined quantity of collagen suspension was centrifuged at 1700× *g* for 5 min, then resuspended in 0.2 M Na_2_HPO_4_ buffer solution (pH 7.5; Sigma-Aldrich, Merck KGaA, Darmstadt, Germany) to reach a final concentration of 5 mg/mL. To each milliliter of this suspension, 0.129 mL of methacrylic anhydride (MA, 94% purity, stabilized with ~0.2% 2,4-dimethyl-6-tert-butylphenol; Thermo Fisher Scientific, Waltham, MA, USA) was added. The reaction was then stirred magnetically in the dark at room temperature for 6 h.

The resulting product was dialysed (cellulose membrane tubing, Sigma-Aldrich, Merck KGaA, Darmstadt, Germany) against distilled water for 10 days at 4 °C. This was followed by multiple washes with distilled water to completely remove any unreacted MA, buffer residues, and by-products.

The methacrylated collagen was then centrifuged at 1700× *g* for 5 min and resuspended in distilled water to obtain a solution with a concentration of 10 mg/mL. Irgacure 2959 photoinitiator (methanol solution; Sigma-Aldrich, Merck KGaA, Darmstadt, Germany) was then added at a concentration of 10% *w*/*w* relative to the collagen content. One milliliter of the resulting mixture was placed in a 24-well Petri dish and irradiated with UV light (365 nm) for 30 min (UV Exposure System, UV lamp Model No. KGW9N PL Lamp 9 W × 4 EA; Edmund Optics, Barrington, NJ, USA) to ensure complete hydrogel formation and crosslinking. The hydrogels (CollMA) were then frozen at −80 °C and lyophilised overnight (Edwards Pirani 1001, Edward Vacuum, Burgess Hill, UK) prior to use.

Control collagen scaffolds (Coll) were produced by centrifuging sea urchin collagen at 1700× *g* for 5 min, then resuspending it in a 6% (*v*/*v*) ethanol/water solution to obtain a 10 mg/mL suspension [12]. One milliliter of this collagen suspension was poured into rubber silicone molds with a diameter of 1 cm. The samples were frozen at −80 °C overnight and freeze-dried (Edwards Pirani 1001).

To characterize the internal morphology of both scaffolds, observations and image acquisition of their ultrastructure were carried out using a field-emission scanning electron microscope (FE-SEM Sigma, ZEISS, Oberkochen, Germany). Briefly, the cross sections of freeze-dried hydrogels and scaffolds were fixed onto aluminum stubs, sputter-coated with a thin gold layer (Leica ACE600, Leica Microsystems, Wetzlar, Germany), and imaged for morphological visual comparison.

### 2.4. Cell Culture Maintenance

C2C12 mouse adherent myoblasts (AddexBio, San Diego, CA, USA) were grown in complete Dulbecco’s modified Eagle’s medium (DMEM; Sigma-Aldrich, Merck KGaA, Darmstadt, Germany) supplemented with 10% fetal bovine serum (FBS; Sigma-Aldrich, Merck KGaA, Darmstadt, Germany). The cell line was maintained in a 5% CO_2_ atmosphere at 37 °C. To induce myogenic differentiation, when cells reached about 80% cell confluence, the medium containing 10% FBS was substituted with 2% horse serum (Sigma-Aldrich, Merck KGaA, Darmstadt, Germany).

### 2.5. Recellularization of Marine Collagen-Based Scaffolds

Two different types of scaffolds were placed into the 8-well chamber slide with a 0.8 cm^2^ growth area. 1 × 10^5^ C2C12 cells in 200 μL complete medium were placed/injected with a Hamilton syringe (Hamilton Central Europe S.R.L., Giarmata, Romania) onto the Coll scaffold or into the CollMA scaffold and allowed to infiltrate in the incubator for 1 h. After 1 h, 200 μL of complete medium was added.

### 2.6. C2C12 Cells Live Imaging on the Surface of the Scaffold

For live imaging, CellTracker (1 mM final concentration, Orange CMRA C34551, ThermoFisher Scientific, Waltham, MA, USA) was used for the Coll scaffold according to the manufacturer’s instructions. Cells were scanned with a Leica Thunder Imager DMi8 microscope (Leica Microsystems, Wetzlar, Germany).

### 2.7. AlamarBlue Assay: C2C12 Viability

The metabolic activity of C2C12 seeded onto biomaterials, as previously described, was assessed by alamarBlue™ Cell Viability Reagent (ThermoFisher Scientific, Waltham, MA, USA) at 1, 4, and 8 days. alamarBlue™ stock solution was diluted to 10% (*v*/*v*) in cell culture medium and kept in the dark.

At each time point, medium was removed, and biomaterials were moved to a new well. Then, biomaterials were washed twice with PBS, and 500 μL of alamarBlue™ solution was added to each well. Biomaterials were incubated for 4 h in an incubator at 37 °C and 5% CO_2_ and protected from light. Then, 100 μL of supernatant solution was aliquoted into a 96-well culture plate for a total of four times. The fluorescent signal was read using a microplate reader (Varioskan LUX Multimode Microplate Reader, ThermoFisher Scientific, Waltham, MA, USA) at λ = 544/590 nm (ex/em). Biomaterials without cells were used as blank samples, and C2C12 seeded onto plastic were used as positive controls. The experiment was repeated at least three times (*n* = 12).

### 2.8. Histological Cell Infiltration Depth Analysis of C2C12 Cells

Scaffolds with cells were fixed at different time points in buffered neutral paraformaldehyde at 4 °C, washed in phosphate-buffered saline, and dehydrated through a graded series of ethanol. Samples embedded in paraffin were cut at 5 μm and stained with hematoxylin and eosin (H&E). Sections were clarified and mounted in Eukitt balsam (ORSA-tec, Bobingen, Germany) for microscopic examination. The images were acquired by the automated slide scanner (Axioscan 7, Zeiss, Germany) and analyzed with OMERO (version 5.19.0. Open Microscope Environment).

### 2.9. Immunofluorescence Analysis

Five μm sections were incubated with primary antibodies anti-MyoD1 (1:500, 18943-1-AP, Proteintech, Wuhan, China) or anti-Pax7 (20570-1-AP, 1:500, Proteintech, Wuhan, China) and subsequently with Alexa Fluor 568 red or Alexa Fluor 488 green secondary antibody (dilution 2 µg/mL, ThermoFisher Scientific, Waltham, MA, USA), respectively. Nuclear morphology was characterized by staining with Hoechst 33,342 (dilution 1 µg/mL, ThermoFisher Scientific, Waltham, MA, USA). Glass coverslips were coated with Mowiol (Sigma-Aldrich, Merck KGaA, Darmstadt,). Confocal microscopy was performed using a TCS-SP5 II confocal laser scanning microscope (Leica Microsystems, Wetzlar, Germany).

### 2.10. Statistical Analysis

Data were expressed as mean ± standard deviation (SD). Statistical analyses were performed using the one-way ANOVA test followed by Tukey’s “post hoc” test for cell viability, whereas cell infiltration within the biomaterial was analyzed using a two-way ANOVA, followed by uncorrected Fisher’s LSD “post hoc” tests. GraphPad Prism software (version 8.0.2) was used to perform the analysis. A level of confidence of *p* < 0.05 was used for statistical significance.

## 3. Results

### 3.1. Coll and CollMA Scaffolds Characterization

Two types of 3D scaffolds were prepared starting from a suspension of collagen fibrils extracted from non-processed sea urchin food waste. Based on the different production protocols [9,14] and Materials and Methods, the two 3D porous sponge-like scaffold types are here denoted as Coll [9] and CollMA [14], respectively.

**Figure 1:**
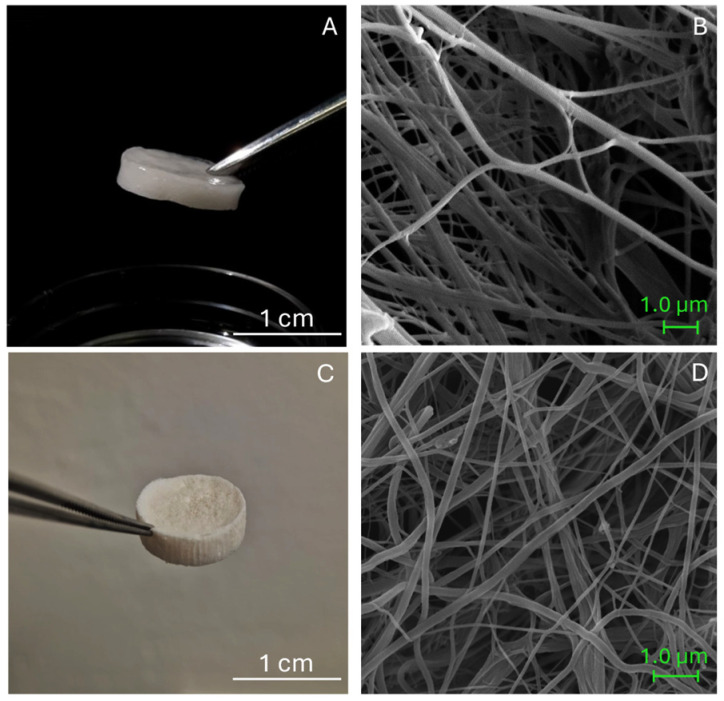
(**A**) CollMA biomaterial macroscopic appearance after hydration; (**B**) SEM image of CollMA. (**C**) Coll biomaterial macroscopic appearance before hydration; (**D**) SEM image of Coll.

After production, both biomaterials were characterized by a porous sponge-like and ECM-mimicking structure (Figure 1A,C), composed of a complex network of long collagen fibrils (Figure 1B,D). The ultrastructure of Coll and CollMA was examined by scanning electron microscopy (SEM) to investigate the fibrillar network. Both biomaterials displayed a homogenous fibrillar structure with no visible aggregates (Figure 1B,D). Coll and CollMA were previously characterized morphologically in a separate study by Roncoroni et al. [14], in which differences in macroporosity, swelling behavior upon hydration, water uptake, and degradation kinetics under physiological and enzymatic conditions were also evaluated. To assess the ability to be used as platforms for the generation of a 3D skeletal muscle tissue model, Coll and CollMA were seeded with the C2C12 murine muscle cell line at the same density.

### 3.2. C2C12 Cell Line Viability

**Figure 2:**
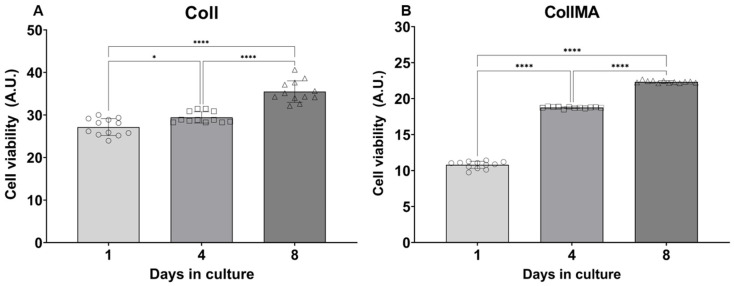
C2C12 metabolic activity when co-cultured with (**A**) Coll or (**B**) CollMA biomaterial. Data are expressed as arbitrary units (A.U.) and as mean ± SD; data were analyzed by one-way ANOVA followed by “post hoc” Tukey’s test; * *p* < 0.05, **** *p* < 0.0001 (*n* = 12 per time point).

Sea urchin-derived collagen has already been proven to be cytocompatible in fibroblast cultures, including primary human dermal fibroblasts [9,10] as well as horse mesenchymal stem cells [12]. In this work, cytocompatibility testing was conducted employing the C2C12 myoblast cell line. The metabolic activity of C2C12 seeded onto Coll or CollMA was quantitatively evaluated by the alamarBlue™ assay at different days of culture as previously described [10,11] (Figure 2). In both culture conditions, a progressive increase in fluorescence intensity was observed, with the highest intensity reported at the endpoint of culture (Day 8). Differences in intensity were markedly observed when cells were co-cultured with CollMA; in this culture setting, cells showed a statistically significant increase in fluorescence at each time point (*p* < 0.0001). Similarly, when C2C12 were seeded onto Coll scaffolds, an increasing fluorescence was observed throughout the experimental period with statistical differences among all time points (Day 1 vs. Day 4: p 0.0241; Day 1 vs. Day 8: *p* < 0.0001; Day 4 vs. Day 8: *p* < 0.0001). Overall, both biomaterials showed cytocompatibility for C2C12 cells, as co-cultured cells showed an increased metabolic activity throughout the experimental period, with higher values observed when co-cultured with Coll.

### 3.3. Infiltration Analyses of C2C12 Cells Within the Coll and CollMA Scaffolds

**Figure 3:**
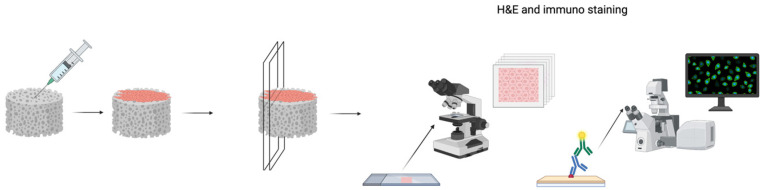
Graphical explanation of infiltration analysis. Coll and CollMA were seeded with the C2C12 myoblast cell line. After the different time points post-seeding, scaffolds were fixed, longitudinally sectioned into 5 μm slices, and stained using an adequate method for cell infiltration analyses. Created in BioRender. Akyurek, E. (2026) https://BioRender.com/mnwchi8 (accessed on 21 December 2025).

**Figure 4:**
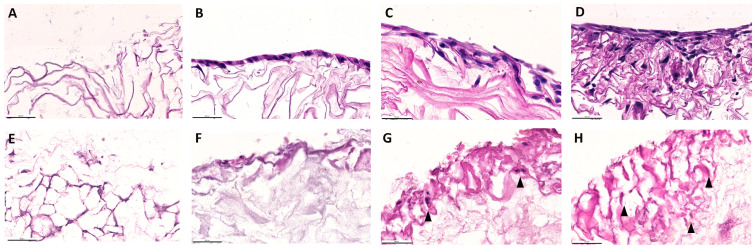
Cell infiltration analysis of Coll (**A**–**D**) and CollMA (**E**–**H**) scaffolds. H&E-stained histological sections were used to evaluate cell infiltration at various time points post-seeding. Unseeded Coll (**A**) and CollMA (**E**) scaffolds were compared with samples collected on day 4 (**B**,**F**), day 6 (**C**), and day 8 (**D**,**G**,**H**) to assess the progression of cellular ingrowth. Arrowheads indicate cells and their nuclei. Images were acquired at identical magnification, with the scale bars representing 50 μm.

The Coll and CollMA scaffolds were placed in chamber slides, and C2C12 myoblasts were seeded onto the surface. The adhesion and infiltration of cells were evaluated at three time points post-seeding (days 4, 6, and 8). At each time point, specimens were fixed and embedded in paraffin. Then, samples were cut longitudinally from top to bottom (Figure 3) and stained with H&E (Figure 4) to visualize cellular components within Coll and CollMA.

On day 4, cells were predominantly observed as a layer on the surface of both Coll and CollMA scaffolds (Figure 4B,F). Live imaging analysis of the scaffold upper surface confirmed that C2C12 established a layer reaching approximately 80% confluence, indicative of a high surface cell density, and exhibited an elongated myoblast morphology (Figure S1), consistent with a previous report [15]. As the culture period progressed (days 6 and 8), C2C12 cells gradually infiltrated throughout both scaffold types, establishing a multilayered cellular population (Figure 4C). By day 8, a dense cellular layer was evidenced throughout both Coll and CollMA scaffolds (Figure 4D,G,H).

**Figure 5:**
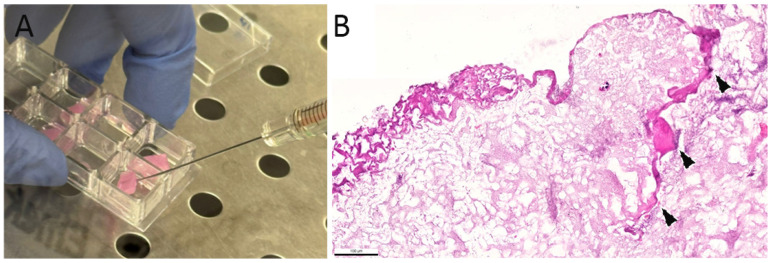
Cell seeding and infiltration in CollMA scaffolds. (**A**). Representative image of cell seeding into the CollMA scaffold using a Hamilton syringe. (**B**). Arrowheads indicate C2C12 cells deposited within the scaffold matrix 8 days after syringe-mediated seeding. Scale bar: 100 μm.

As reported by Ferrario et al. [10], the Coll scaffold displays a structurally intact surface, characterized by well-defined laminar architectures, which remain continuous in both dry and hydrated conditions, including when wetted with cell culture medium. In contrast, once hydrated, the CollMA scaffold acquires a markedly softer consistency and a semi-transparent appearance, indicative of its higher water uptake and increased matrix compliance. These morphological features allow the direct seeding of cells into the scaffold using a Hamilton syringe, not only on the surface, which may contribute to a more homogeneous distribution across the entire matrix (as shown in Figure 5).

### 3.4. Quantitative Evaluation of Cell Infiltration of C2C12 Cells into the Coll and CollMA Scaffolds

**Figure 6:**
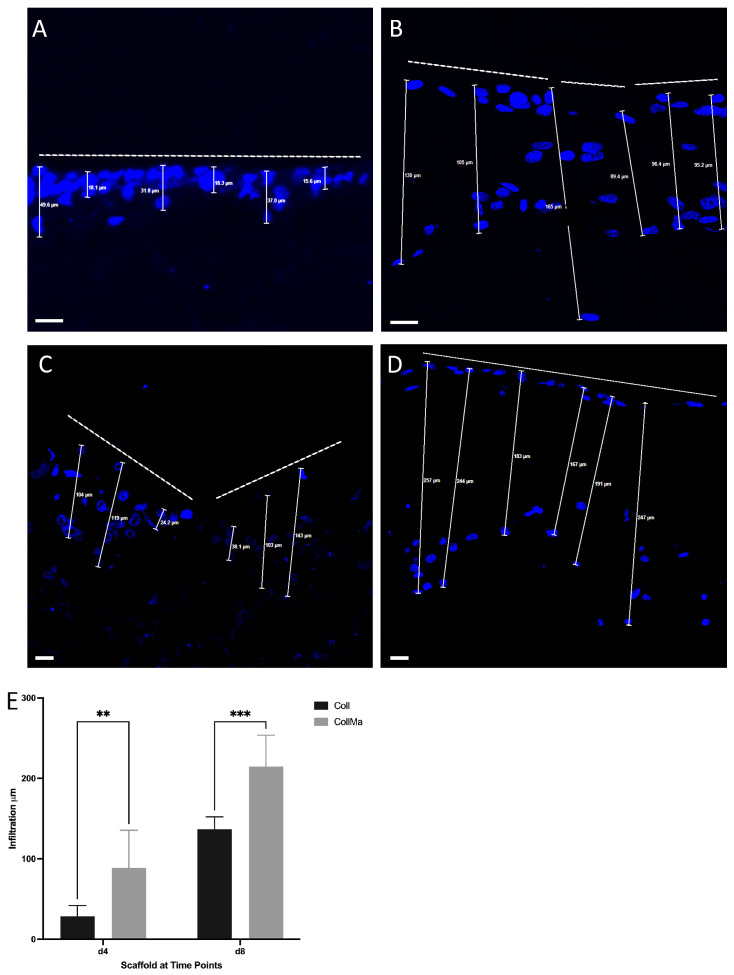
Cell infiltration depth analysis in Coll (**A**,**B**) and CollMA (**C**,**D**) scaffolds. Five-μm-thick sections of Coll and CollMA scaffolds were stained with Hoechst to detect and evaluate cell infiltration depth at different time points. Representative images are shown for day 4 (**A**,**C**), and day 8 (**B**,**D**). The white dashed lines delineate the boundary of the scaffold surface where cells were initially seeded. Scale bars: 20 μm. Quantitative comparison of infiltration depth in Coll and CollMA scaffolds across time points is shown in the graph (**E**). Statistical analysis was performed using a two-way ANOVA test. Data are expressed as mean ± SD and were obtained from six biological replicates (*n* = 6 per time point). ** *p* < 0.01, *** *p* < 0.001.

The Coll and CollMA scaffolds, placed in chamber slides, were seeded onto the surface with C2C12 myoblasts, and cell infiltration was evaluated by examining the spatial distribution of nuclei within the scaffolds. To this aim, 5 μm-thick sections of both Coll and CollMA fixed specimens were counterstained with Hoechst to enable nuclear detection and subsequently imaged using high-resolution confocal microscopy. The infiltration depth was quantified as the linear distance between the scaffold surface and the position of the C2C12 cell, identified by its nucleus within the matrix, as depicted in Figure 6 and Figure S2. Measurements, expressed in micrometers, demonstrated that cell infiltration depth within the CollMA scaffold was significantly greater than that observed in the Coll scaffold at corresponding time points (Figure 6E).

### 3.5. Analysis of the C2C12 Myoblast Differentiation Within CollMA Scaffold

Given the observation that C2C12 myoblasts exhibited enhanced infiltration depth within the CollMA scaffold and its porous microarchitecture enabled direct cell seeding into the interior matrix, we investigated the early stages of C2C12 differentiation within the CollMA scaffold microenvironment.

During myogenic differentiation, C2C12 myoblasts undergo coordinated morphological and molecular changes driven by the regulated activity of myogenic regulatory factors (MRFs). MyoD displays a biphasic expression pattern, supporting both myoblast commitment and terminal differentiation [16,17]. Conversely, Pax7, which sustains satellite cell maintenance and myoblast proliferation, is progressively downregulated upon differentiation onset, thereby enabling myogenic progression and myotube formation [18].

Longitudinal sections from the CollMA scaffold at 4 and 8 days post-seeding were immunolabeled with either anti-Pax7 or anti-MyoD primary antibodies and counterstained with Hoechst to visualize nuclei.

**Figure 7:**
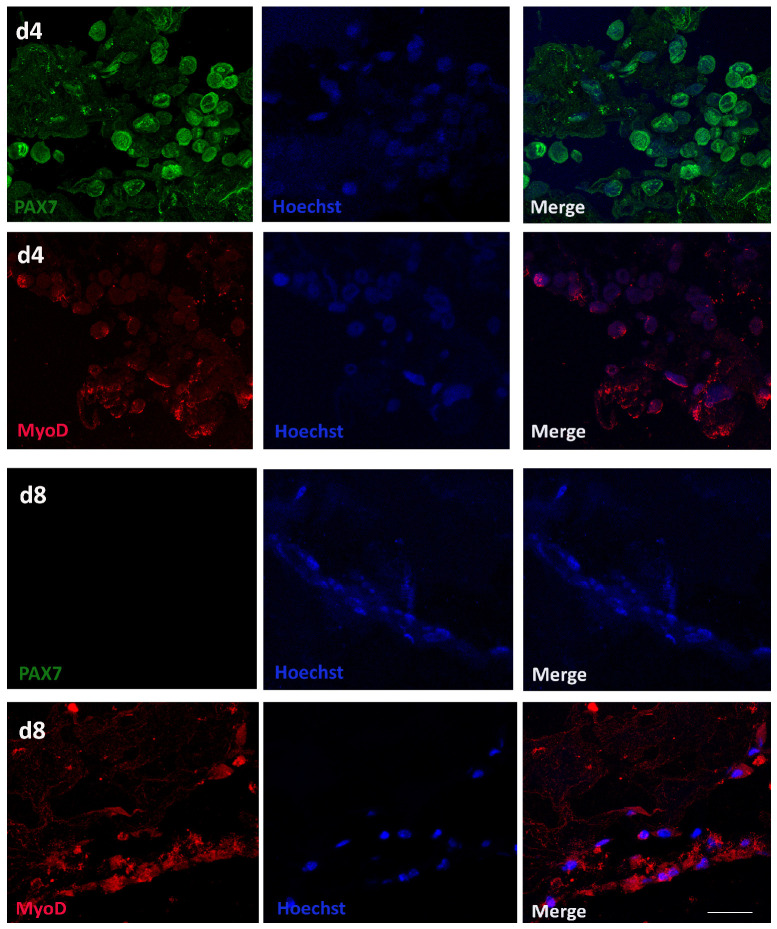
C2C12 myoblast differentiation within CollMA scaffold microenvironment. Five-μm-thick sections of CollMA scaffolds were stained at day 4 (d4) with Pax7 (green) and MyoD (red) antibodies to identify C2C12 myoblasts. After the differentiation induction, 5-μm-thick sections of CollMA scaffolds were stained on day 8 (d8) with Pax7 (green) and MyoD (red) antibodies to assess the myogenic differentiation stage of C2C12 cells. Hoechst (blue) was used to visualize nuclei. Scale bar: 30 μm.

Prior to differentiation induction, as depicted in Figure 7, cells at time point day 4 exhibited co-expression of Pax7 (green) and MyoD (red), indicative of their proliferative myoblast state. Conversely, on day 8, Pax7 immunoreactivity was markedly lower, suggesting induction of cell differentiation, while robust MyoD expression persisted (Figure 7).

## 4. Discussion

In this study, we propose two 3D skeletal muscle collagen-based culture systems, designed to recapitulate the skeletal muscle tissue and act as an alternative model to in vivo studies.

Skeletal muscle is one of the most widely distributed tissues in mammals and other vertebrate species, comprising approximately 40% of total body mass in mammals [19].

Skeletal muscle exhibits a highly organized structure consisting of bundles of cells called muscle fibers. Each muscle is enclosed by the outermost layer of connective tissue, the epimysium. Bundles of muscle fibers are held together by a continuous network of intramuscular connective tissue elements known as the perimysium. Finally, a thin layer of intramuscular connective tissue that resembles a soft gel, termed the endomysium, surrounds and envelopes each individual muscle fiber. This honeycomb-like endomysial network establishes an integrated three-dimensional matrix within the fiber, thereby creating a structural continuity among neighboring muscle fibers [20,21].

Traditional anatomical terms such as endomysium and perimysium were largely used in the past to describe the skeletal muscle ECM [22]. Nowadays, it has been well established that muscle fibers are embedded into an ECM consisting of a mesh-like structure of collagenous components and a mixture of macromolecules, such as proteoglycans and glycoproteins [23]. Collagen represents the principal structural protein of the skeletal muscle ECM [24,25,26]. In vertebrates, up to 28 distinct collagen isoforms have been described [27]. Collagen type I is the isoform predominantly expressed in the endomysium, while type III collagen isoform is more widely represented throughout the perimysium and epimysium [20,28].

In recent years, 3D cell culture systems have emerged as promising tools for muscle research, as they recapitulate key features of muscle tissue architecture and provide an intermediate level of complexity between conventional two-dimensional (2D) monolayer cultures and in vivo animal models. In this context, scaffolds for 3D culture systems play a pivotal role in the development of engineered skeletal muscle tissue. Functional scaffolds should be able to mimic the in vivo ECM not only to support primary cells but also to promote cell adhesion, migration, proliferation, and differentiation.

The main scaffold types employed for skeletal muscle tissue engineering applications are derived from natural or synthetic polymers or by mixing components. Synthetic scaffolds are based on biocompatible polymers, such as poly-L-lactic acid or polyethylene glycol [29]. Polysaccharide-derived biomolecules, with alginate being the most widely employed, are also utilized in skeletal muscle modeling [30]. On the other hand, collagen, laminin [31], and fibrinogen are the biomolecules predominantly utilized for the creation of natural scaffolds [32]. Type I collagen, the predominant form used for skeletal muscle tissue engineering [33], can be extracted from tissues of various organisms, ranging from mammals (bovine, porcine, or equine species) to marine species (sponges, jellyfish, mollusks, and fish). Both natural and synthetic sources exhibit the potential for chemical functionalization, enabling photo-induced crosslinking reactions to yield hydrogel constructs [33].

Collagen is one of the most exploited structural proteins for skeletal muscle tissue engineering purposes [34]. Herein, we described two types of 3D porous sponge-like scaffolds, namely Coll and CollMA, which were prepared starting from a fibrillar collagen suspension extracted from non-processed sea urchin (*Paracentrotus lividus*) food waste. The collagen derives from the peristomal membrane of the animal, which is the soft tissue surrounding the sea urchin’s mouth [12]; concomitantly, the valorization of a food waste by-product makes it a sustainable and eco-friendly strategy for biomaterial production in a circular economy scenario. The main drawback of this approach might be attributed to the lower amount of starting material due to seasonal fishing bans in the Mediterranean area (i.e., scarcity of raw materials in restaurants) during certain periods. Nonetheless, it can be overcome by properly stocking raw material as previously described, making the production of scaffold viable throughout the year [9,10,11,12,14].

Notably, the collagen from the sea urchin peristomal membrane has been shown to closely resemble mammalian type I collagen in chain composition, immunoreactivity, and ultrastructural characteristics [12,35]. Consequently, this collagen appears to be well suited to replicate the ultrastructure of the skeletal muscle endomysium [20]. Furthermore, the collagen extracted from sea urchins emerges as a viable substitute for mammalian-derived collagen and other marine collagen sources, as it exhibits comparable extraction yields while retaining the native conformational integrity of the biomaterial [10].

As reported by Ferrario et al. [10] and in Figure 1D, in the Coll scaffold, collagen fibrils exhibit an irregular and non-oriented arrangement, resulting in a loosely organized fibrous matrix. A similar arrangement is observed in CollMA, as evidenced by the SEM analyses presented here (Figure 1B) and Roncoroni et al. [14]. The disordered distribution contributes to the formation of a porous, mesh-like collagen network that extends throughout the scaffolds.

The ability of these three-dimensional constructs to recapitulate native skeletal muscle tissue architecture and support cell growth was evaluated by seeding the marine collagen scaffolds with the C2C12 myoblast cell line. Establishing the cytocompatibility of engineered scaffolds with cells is a fundamental prerequisite to further progress in biomaterial development and functionalization. Cell viability assays demonstrated that the metabolic activity of C2C12 cells increased on both Coll and CollMA scaffolds, hence a higher number of cells, with the increase being nearly identical between the two scaffolds. These findings are in strong agreement with those reported in earlier studies using the same methodology with dermal fibroblasts cultured on Coll scaffolds [10,11].

It is well known that for optimal functionality, a scaffold should exhibit high porosity to guarantee effective cell infiltration. Upon hydration, both Coll and CollMA exhibited macroporosity above 80%, indicating that the majority of the scaffold volume is occupied by interconnected macropores [14]. The internal architecture of Coll and CollMA scaffolds, characterized by a continuous system of interconnected pores of varying sizes and shapes, was shown to facilitate C2C12 cell migration into the thick matrix. Roncoroni et al. [14] also reported that the two biomaterials exhibited different swelling behaviors upon hydration: CollMA increased in thickness (+29.19% ± 0.22) with a moderate area reduction (−16.40% ± 0.03), while Coll collapsed, with a thickness decrease of −73.10% ± 0.09 and area expansion of +39.00% ± 0.15. Although both materials absorbed large amounts of water, water uptake was markedly lower in CollMA (1064% ± 58) than in Coll (11,340% ± 755), confirming the stabilizing effect of methacrylation. In the CollMA scaffold, the highly stable structure facilitates cellular infiltration under identical incubation and culture conditions. Furthermore, upon hydration, the soft architecture of CollMA scaffolds not only enabled deeper C2C12 cell penetration but also promoted cell distribution throughout the scaffold thickness, a process that can be efficiently achieved by injecting the cell suspension through the center of the scaffold using a syringe.

In this context, the CollMA scaffold is likely to provide a mechanical microenvironment that more closely resembles that of the native skeletal muscle ECM, thereby supporting the transition from proliferative Pax7-positive myogenic progenitors toward MyoD-expressing differentiating cells observed in this study. Notably, the higher macroporosity observed in Coll scaffolds [14] could have provided an optimal environment for cell proliferation (i.e., more freely available space for expansion). Conversely, the “steric hindrance”, or physical confinement, observed in CollMA hydrogel might have likely served as a mechanical cue, triggering the transition towards a differentiating state [36,37]; this would justify the comparatively differential metabolic activity observed between the two constructs.

Indeed, within the CollMA scaffold, by day 8, C2C12 Pax7 immunoreactivity showed an appreciable diminished positivity, reflecting the progression of C2C12 cells towards myogenic commitment. Conversely, MyoD expression remained robust. It has been reported that the ratio between collagen fibrils and water determines the capacity of a scaffold to support efficient nutrient transport, thereby influencing cellular vitality and differentiation within the matrix [38]. This reciprocal shift in Pax7 and MyoD expression, consistent with the transition from proliferating myoblasts to terminally differentiated myotubes, appears to be facilitated by the favorable microenvironment provided by the CollMA scaffold.

## 5. Conclusions

Taken together, our results suggest that sea urchin–derived collagen matrix, especially the CollMA, represents a promising scaffold for skeletal muscle tissue engineering. It has been demonstrated that CollMA cellularized with C2C12 might act as a robust platform for skeletal muscle modeling by supporting cell viability and induction towards differentiation. This system might pave the way for advanced disease modeling for muscle disorders.

Moreover, as the starting material derives from waste generated by the food industry, it ensures low production costs and enhances the economic viability of the process from a circular economy perspective.

Future research should be focused on utilizing this platform for disease modeling, potentially offering a more ethical and cost-effective alternative to animal models. The integration of primary cell culture from healthy or diseased donors, alongside the co-culture of other muscle-resident cell populations (e.g., motoneurons), and the addition of physiological stimuli, will be fundamental to enable a functional maturation of these constructs. This might enable the generation of an in vitro preclinical platform suitable for evaluating pharmacological compounds for precision or personalized therapy.

## Data Availability

The data presented in this study are available on request from the corresponding author.

## Supplementary Materials

The following supporting information can be downloaded at: https://www.mdpi.com/article/10.3390/ani16030512/s1, Figure S1: live imaging of C2C12 cells on the standard scaffold surface. Figure S2: Cell infiltration depth analysis in Coll (A,B) and CollMA (C,D) scaffolds as in Figure 6.

## Abbreviations

The following abbreviations are used in this manuscript:

ECM	Extracellular matrix
Coll	Standard collagen scaffold
CollMA	Methacrylated collagen scaffold/hydrogel
