# Supplementary material for: Collagen-Based 3D Scaffolds from Sea Urchin Food Waste for Skeletal Muscle Tissue Engineering
Source: Animals (Basel). 2026 Feb 5;16(3):512. doi: 10.3390/ani16030512 (PMC12896538; doi:10.3390/ani16030512)
Supplement: Supplementary file 1 [file animals-16-00512-s001.zip › animals-4087184-supplementary.pdf]

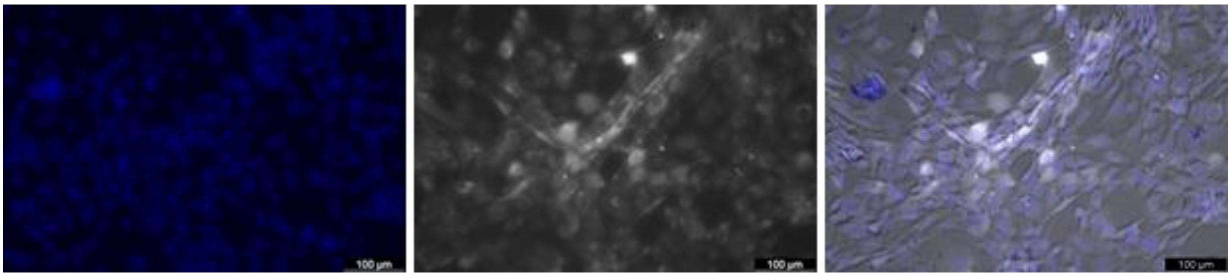

Supplementary Figure S1. Live imaging of C2C12 cells on the Coll scaffold surface. C2C12 cells labelled with CellTracker™ Orange Fluorescent Probe (white) for tracking on the scaffold surface. Cell nuclei were stained (bleu) with Hoechst. The merged image combines bright-field view with CellTracker and Hoechst fluorescence, providing comprehensive visualization of cell morphology and distribution. Scale bar: 100 µm.

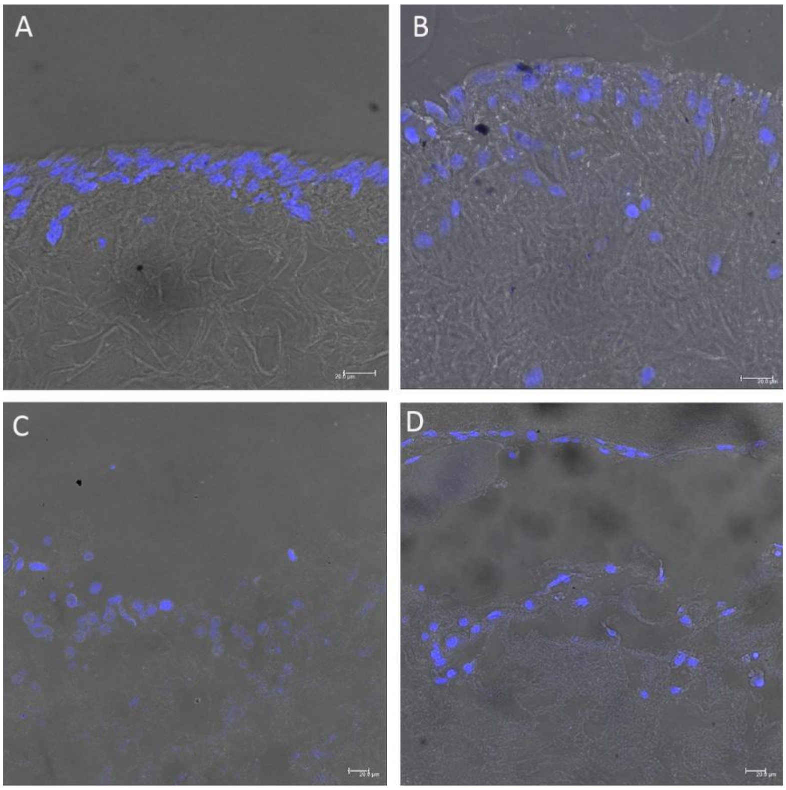

Supplementary Figure S2. Cell infiltration depth analysis in Coll (A, B) and CollMA (C, D) scaffolds as in Figure 6. Representative images of day 4 (A, C) and day 8 (B, D) showing the overlay between bright-fields and Hoechst nuclear staining. Scale bars 20 µm.
